# 2-Deoxy-D-glucose enhances TRAIL-induced apoptosis in human melanoma cells through XBP-1-mediated up-regulation of TRAIL-R2

**DOI:** 10.1186/1476-4598-8-122

**Published:** 2009-12-14

**Authors:** Hao Liu, Chen Chen Jiang, Christopher J Lavis, Amanda Croft, Li Dong, Hsin-Yi Tseng, Fan Yang, Kwang Hong Tay, Peter Hersey, Xu Dong Zhang

**Affiliations:** 1 Immunology and Oncology Unit, Room 443, Calvary Mater Newcastle Hospital, NSW, Australia; 2 Faculty of Pharmacy, Bengbu Medical College, Bengbu, Anhui, PR China

## Abstract

**Background:**

Past studies have shown that sensitivity of melanoma cells to TRAIL-induced apoptosis is largely correlated with the expression levels of TRAIL death receptors on the cell surface. However, fresh melanoma isolates and melanoma tissue sections express generally low levels of death receptors for TRAIL. The clinical potential of TRAIL in the treatment of melanoma may therefore be limited unless given with agents that increase the cell surface expression of TRAIL death receptors. 2-Deoxy-D-glucose (2-DG) is a synthetic glucose analogue that inhibits glycolysis and glycosylation and blocks cell growth. It has been in clinical evaluation for its potential use as an anticancer agent. In this study, we have examined whether 2-DG and TRAIL interact to enhance their cytotoxicity towards melanoma cells.

**Results:**

2-DG did not kill melanoma cells, but enhanced TRAIL-induced apoptosis in cultured melanoma cells and fresh melanoma isolates. This was associated with increased activation of the caspase cascade and mitochondrial apoptotic pathway, and was blocked by inhibition of TRAIL-R2, and to a lesser extent, inhibition of TRAIL-R1. Treatment with 2-DG up-regulated TRAIL death receptors, in particular, TRAIL-R2, on the melanoma cell surface. Up-regulation of TRAIL-R2 was due to increased transcription that was not dependent on the transcription factors, p53 and CHOP. Instead, the IRE1α and ATF6 pathways of the unfolded protein response that were activated by 2-DG appeared to be involved. Moreover, XBP-1, which is known to be transcriptionally regulated by ATF6 and functionally activated by IRE1α, was found to play an important role in 2-DG-mediated transcriptional up-regulation of TRAIL-R2 in melanoma cells.

**Conclusion:**

These results indicate that 2-DG sensitizes human melanoma cells to TRAIL-induced apoptosis by up-regulation of TRAIL-2 via the ATF6/IRE1α/XBP-1 axis of the unfolded protein response. They suggest that 2-DG is a promising agent to increase the therapeutic response to TRAIL in melanoma.

## Introduction

TNF-related apoptosis-inducing ligand (TRAIL) appears to be a promising candidate for cancer therapeutics because of its ability to preferentially induce apoptosis in malignant cells [1-3]. The potential significance of TRAIL as an anti-cancer agent has been supported by studies in animal models showing selective toxicity to human tumor xenografts but not normal tissues [4,5]. Induction of apoptosis by TRAIL is mediated by its interaction with two death domain containing receptors, TRAIL-R1 and -R2 [1-3]. This in turn orchestrates the assembly of the death-inducing signaling complex (DISC) that contains adapter components such as Fas associated death domain (FADD) that activates initiator caspases, caspase-8 and -10, leading eventually to activation of effector caspases such as caspase-3 and to apoptosis [1-3]. TRAIL and agonistic antibodies against its death receptors are currently in clinical evaluation for the treatment of various cancers [6-8].

We have previously shown that sensitivity of cultured melanoma cells to TRAIL-induced apoptosis is in general correlated with the levels of the cell surface expression of TRAIL death receptors, in particular, TRAIL-R2 [9,10]. Subsequent studies demonstrated that fresh melanoma isolates are relatively resistant to TRAIL-induced apoptosis due to low levels of TRAIL-death receptor expression [11]. Moreover, melanoma cells selected for TRAIL resistance by prolonged exposure to TRAIL express substantially reduced levels of TRAIL-R2 on their surface [12,13]. Studies on melanoma tissue sections revealed that reduced TRAIL-R2 expression is associated with disease progression and a poor prognosis [14]. Taken together, these studies indicate that melanoma may not respond to treatment with TRAIL unless given with agents that increase the cell surface expression of TRAIL death receptors, in particular, TRAIL-R2.

Cancer cells exhibit increased glycolysis and depend on this metabolic pathway for ATP production [15-17]. As a consequence, they need a high uptake of glucose and accelerated rates of glycolysis to survive. This metabolic feature has evoked much interest in development of glycolytic inhibitors as potential anticancer agents [16,17]. Among them, 2-Deoxy-D-glucose (2-DG) is a synthetic glucose analogue that is phosphorylated by hexokinase upon transport into cells, but can not be fully metabolized [16-18]. 2-DG-6 phosphate accumulates in cells and interferes with glycolysis primarily by inhibition of phosphorylation of glucose by hexokinase, thus causing a depletion of ATP [16,18]. 2-DG can also cause inhibition of protein glycosylation that induces endoplasmic reticulum (ER) stress and gives rise to activation of the unfolded protein response (UPR) [19,20]. As a single agent, 2-DG has been shown to inhibit cell growth in a number of cancers, and to enhance the therapeutic efficacy of chemotherapeutic drugs in human cancer xenografts [21-23]. On the other hand, 2-DG has been reported to protect cancer cells from death by activation of the Akt and mitogen-activated protein kinase (MAPK) pathways [24].

The cellular response to ER stress, the UPR, consists of three distinct yet coordinated signaling pathways initiated respectively by inositol-requiring transmembrane kinase and endonuclease 1α (IRE1α), activation of transcription factor 6 (ATF6), and protein kinase-like ER kinase (PERK) [25-27]. As an adaptive response, the UPR is orchestrated by transcriptional activation of multiple genes mediated by IRE1α and ATF6, and a general decrease in translation initiation mediated by PERK, to alleviate the stress condition [25-27]. However, excessive and prolonged activation of the UPR can lead to apoptosis [25-27]. We have previously shown that, although melanoma cells are not sensitive to ER stress-induced apoptosis, activation of the UPR by the glycosylation inhibitor tunicamycin (TM), or the ER Ca^2+ ^ATPases inhibitor thapsigargin (TG), up-regulates TRAIL-R2 and enhances TRAIL-induced apoptosis in melanoma cells [28-30].

In view of the potential application of 2-DG and TRAIL in the treatment of melanoma, we have examined whether they interact to enhance their toxic effect on melanoma cells. We show in this report that the combination of 2-DG and TRAIL enhanced TRAIL-induced apoptosis in melanoma cell lines and fresh melanoma isolates. This was primarily due to up-regulation of TRAIL death receptors, in particular, TRAIL-R2 on the melanoma cell surface. Moreover, we demonstrate that up-regulation of TRAIL-R2 by 2-DG was due to an increase in transcription, but this is not mediated by p53 or CCAAT/enhancer-binding protein-homologous protein (CHOP). Instead, the XBP-1 pathway of the UPR plays an important role in 2-DG-mediated up-regulation of TRAIL-R2 in melanoma cells.

## Results

### 2-DG sensitizes melanoma cells to TRAIL-induced apoptosis

**Figure 1:**
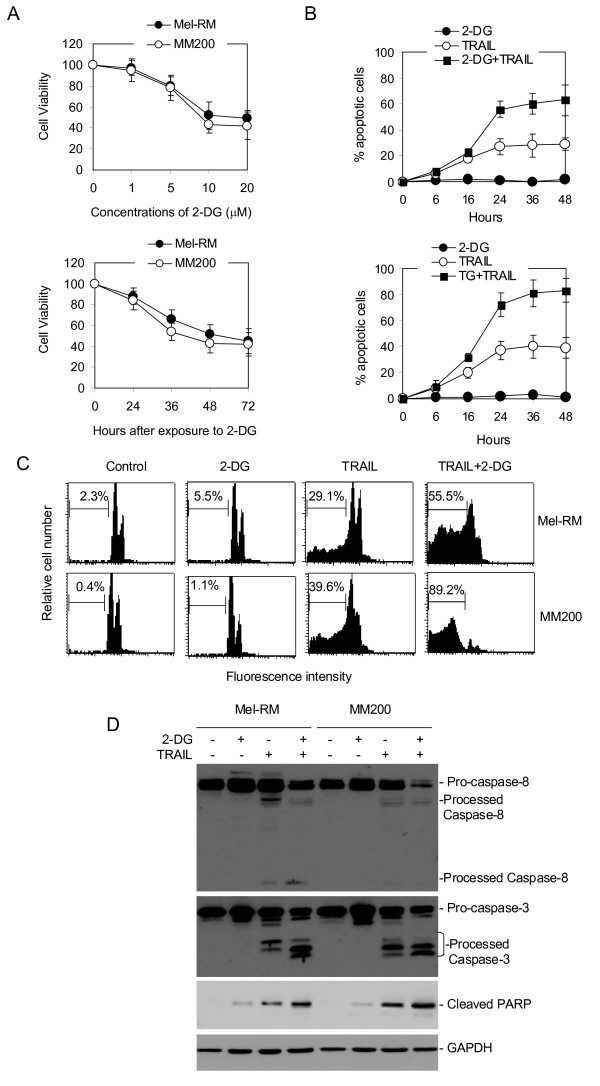
**2-DG sensitizes melanoma cells to TRAIL-induced apoptosis**. **A**, 2-DG inhibits melanoma cell growth. Upper panel: Mel-RM and MM200 cells were treated with 2-DG at indicated concentrations for 48 hours. Cell viability was measured by MTT assays. Lower panel: Mel-RM and MM200 cells treated with 2-DG (10 μM) for indicated periods were subjected to measurement of viability using MTT assays. **B**, Mel-RM (upper panel) and MM200 (lower panel) cells were co-treated with 2-DG (10 μM) and TRAIL (200 ng/ml) for indicated periods before apoptosis was measured by the propidium iodide method using flow cytometry. **C**, Representative flow cytometry histograms of apoptosis assays. Mel-RM and MM200 cells were treated with 2-DG (10 μM), TRAIL (200 ng/ml), or the combination of both for 24 hours. Apoptosis was measured by the propidium iodide method using flow cytometry. **D**, Whole cell lysates from Mel-RM and MM200 cells treated with the combination of 2-DG (10 μM) and TRAIL (200 ng/ml) for 16 hours were subjected to Western blot analysis. Note that PARP was detected by an Ab that specifically recognizes the cleaved form of PARP.

**Figure 2:**
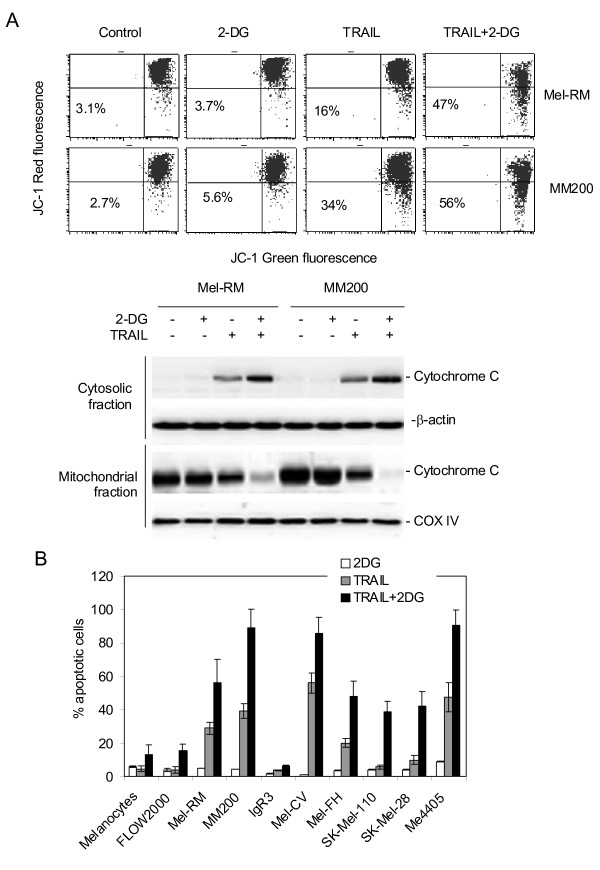
**A, 2-DG enhances activation of the mitochondrial apoptotic pathway by TRAIL**. Upper panel: Mel-RM and MM200 cells treated with the combination of 2-DG (10 μM) and TRAIL (200 ng/ml) for 16 hours were subjected to measurement of ΔΨm by JC-1 staining in flow cytometry. The number in each left bottom quadrant represents the percentage of cells with reduction in ΔΨm. Lower panel: Cytosolic and mitochondrial fractions of Mel-RM and MM200 cells treated with the combination of 2-DG (10 μM) and TRAIL (200 ng/ml) for 16 hours were subjected to Western blot analysis. Western blot analysis of COX IV or β-actin levels was included to show relative purity of the mitochondrial or cytosolic fractions. **B, A summary of studies of the effect of 2-DG on TRAIL-induced apoptosis in a panel of melanoma cell lines and a melanocyte line**. Cells were treated with 2-DG (10 μM) and TRAIL (200 ng/ml) for 24 hours before apoptosis was measured by the propidium iodide method using flow cytometry. The data shown are either the mean ± SE (A, B, & F), or representative (C, D, & E), of three individual experiments.

Our initial studies on two melanoma cell lines, Mel-RM and MM200, indicated that 2-DG alone did not induce notable apoptosis, although it inhibited cell proliferation (Figures 1A &1B). Nevertheless, studies on its effect on TRAIL-induced apoptosis showed that the combination of 2-DG and TRAIL enhanced sensitivity of the cells to apoptosis-induced by TRAIL (Figures 1B &1C). The increase in TRAIL-induced apoptosis in the presence of 2-DG was observed as early as 16 hours and reached a peak at 36 hours after treatment (Figure 1B). In association with this, co-treatment with 2-DG enhanced TRAIL-induced activation of caspase-8, reduction in ΔΨm, mitochondrial release of cytochrome c, activation of caspase-3 and cleavage of its substrate PARP (Figures 1D & Figure 2A). It is of note that the cleaved products of caspase-8 were hardly detected in MM200 presumably due to relatively low concentrations within the cells (Figure 1D). Increased activation of caspase-3 was shown by both decreased cleavage of the pro-enzyme of caspase-3, and reduced conversion of the larger cleaved fragment to smaller ones (Figure 1D).

A summary of studies on the effect of 2-DG on TRAIL-induced apoptosis in a panel of melanoma cell lines and cultured melanocytes and fibroblasts is shown in Figure 2B. As expected, co-treatment with 2-DG enhanced TRAIL-induced apoptosis in all the melanoma lines (p < 0.05). Neither TRAIL nor 2-DG alone induced apoptosis in melanocytes and fibroblasts, but the combination of TRAIL and 2-DG resulted in an increase in apoptosis in both types of normal cells, even though the overall levels of apoptosis remained low (< 20%) (Figure 2B).

### 2-DG up-regulates TRAIL-R2 in melanoma cells

**Figure 3:**
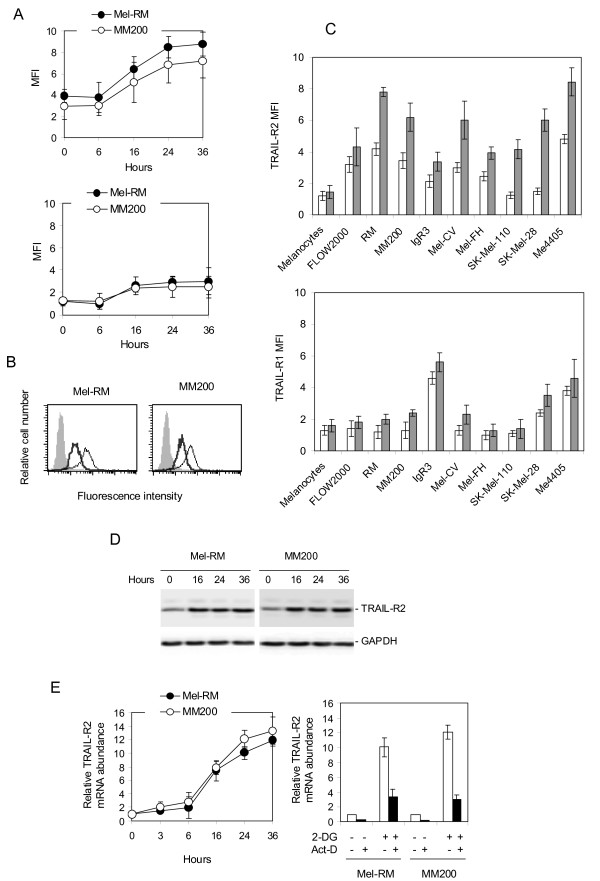
**2-DG up-regulates TRAIL death receptors in melanoma cells**. **A**, Mel-RM and MM200 cells treated with 2-DG (10 μM) for indicated periods were subjected to measurement of the cell surface expression of TRAIL-R2 (upper panel) and -R1 (lower panel) using flow cytometry. The data in y axes represent mean fluorescence intensity (MFI). **B**, Representative flow cytometry histograms showing up-regulation of TRAIL-R2 by 2-DG in melanoma cells. Filled histograms: isotype controls; Thick open histograms: TRAIL-R2 expression before treatment; Thin open histograms: TRAIL-R2 expression after treatment with 2-DG (10 μM) for 24 hours. **C**, 2-DG up-regulates TRAIL-R2 and -R1 in a panel of melanoma cell lines, melanocytes, and fibroblasts. Cells were treated with 2-DG (10 μM) for 24 hours. The cell surface expression of TRAIL-R2 (upper panel), and -R1 (lower panel) was measured using flow cytometry. The data in the y axes represent mean fluorescence intensity (MFI). **D**, Whole cell lysates from Mel-RM and MM200 cells treated with 2-DG (10 μM) for indicated periods were subjected to Western blot analysis. **E**, Left panel: Mel-RM and MM200 cells were treated with the 2-DG (10 μM) for indicated periods. Total RNA was isolated and subjected to Real-time PCR analysis for TRAIL-R2 mRNA expression. The relative abundance of mRNA expression before treatment was arbitrarily designated as 1. The increases in TRAIL-R2 mRNA at 16, 24, and 36 hours after treatment in both cell lines were statistically significant (p < 0.05); Right panel: Mel-RM and MM200 cells were treated with actinomycin D (3 μg/ml) for 1 hour before the addition of 2-DG (10 μM) and TRAIL (200 ng/ml) for a further 24 hours. Total RNA was isolated and subjected to Real-time PCR analysis for TRAIL-R2 mRNA expression. The relative abundance of mRNA expression before treatment was arbitrarily designated as 1. Up-regulation of TRAIL-R2 mRNA by 2-DG was significantly inhibited by actinomycin D in both cell lines (p < 0.05). The data shown are either the mean ± SE (A, C, & E), or representative (B & D), of three individual experiments.

Having found that 2-DG enhances TRAIL-induced activation of caspase-8 (Figure 1D), we examined whether it regulates the cell surface expression of TRAIL receptors in melanoma cells. As shown in Figures 3A &3B, 2-DG up-regulated the expression of TRAIL-R2 on the surface of Mel-RM and MM200 cells, with a significant increase being detected at 16 hours, and further increases at 24 and 36 hours after exposure to the compound. The levels of TRAIL-R1 on the cell surface were also increased by 2-DG, albeit to a lesser extent, in both cell lines (Figure 3A). In contrast, 2-DG did not induce any change in the expression of TRAIL-R3 and -4 on the cell surface (data not shown). Up-regulation of the cell surface expression of TRAIL death receptors by 2-DG was confirmed in a panel of melanoma cell lines (Figure 3C). Treatment with 2-DG resulted in slight increases in TRAIL-R2 and -R1 on the surface of melanocytes and fibroblasts (Figure 3C).

TRAIL-induced apoptosis of melanoma cells is primarily correlated with the levels of TRAIL-R2 expression on the cell surface [9,10]. We therefore focused on investigation of the mechanism by which TRAIL-R2 is up-regulated by 2-DG. To this end, we examined if 2-DG regulates TRAIL-R2 total protein and mRNA levels by Western blotting and Real time PCR, respectively. As shown in Figure 3D, 2-DG increased the levels of the TRAIL-R2 total protein that could be detected by 16 hours after treatment. Figure 3E shows that treatment with 2-DG up-regulated the levels of TRAIL-R2 mRNA in both cell lines. The increase in TRAIL-R2 mRNA levels induced by 2-DG could be inhibited by pretreatment with actinomycin D (Figure 3E), suggesting that this was due to a transcriptional increase, rather than a change in the mRNA stability. Taken together, these results suggest that up-regulation of the cell surface expression of TRAIL-R2 by 2-DG results from increased TRAIL-R2 transcription in melanoma cells.

### Sensitization of melanoma cells to TRAIL-induced apoptosis by 2-DG is largely mediated by up-regulation of TRAIL-R2

**Figure 4:**
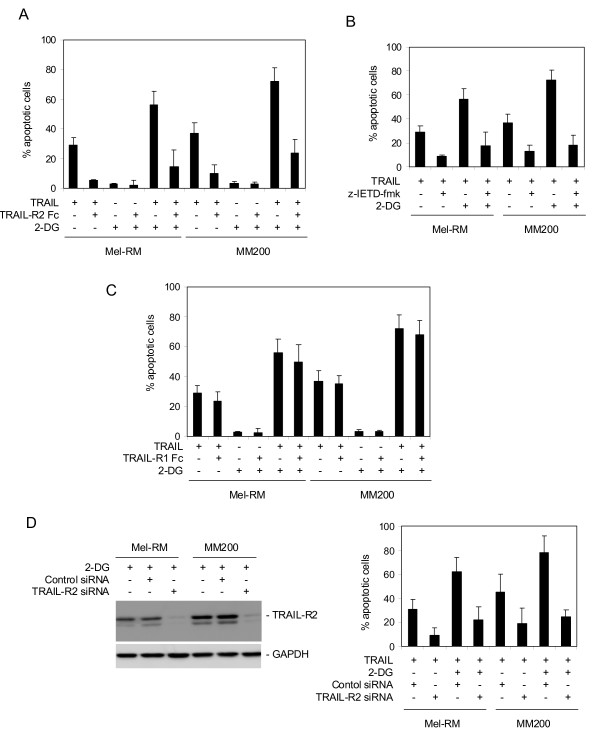
**Sensitization of melanoma cells to TRAIL-induced apoptosis by 2-DG is largely due to up-regulation of TRAIL-R2**. **A**, Mel-RM and MM200 cells were treated with a TRAIL-R2/Fc chimera (10 μg/ml) before the addition of 2-DG (10 μM) and TRAIL (200 ng/ml) for a further 24 hours. Apoptosis was measured by the propidium iodide method using flow cytometry. **B**, Mel-RM and M200 cells were treated the caspase-8 specific inhibitor z-IETD-fmk (30 μM) for 1 hour before the addition of 2-DG (10 μM) and TRAIL (200 ng/ml) for a further 24 hours. Apoptosis was measured by the propidium iodide method using flow cytometry. **C**, Mel-RM and MM200 cells were treated with a TRAIL-R1/Fc chimera (10 μg/ml) before the addition of 2-DG (10 μM) and TRAIL (200 ng/ml) for a further 24 hours. Apoptosis was measured by the propidium iodide method using flow cytometry. **D**, Mel-RM and MM200 cells were transfected with the control or TRAIL-R2 siRNA. Left panel: Twenty-four hours later, whole cell lysates were subjected to Western blot analysis. Right panel: Twenty-four hours later, the cells were treated with 2-DG (10 μM) and TRAIL (200 ng/ml) for a further 24 hours. Apoptosis was measured by the propidium iodide method using flow cytometry. The data shown are either the mean ± SE (A, B, C, & the right panel of D), or representative (the left panel of D), of three individual experiments.

The role of up-regulation of TRAIL-R2 in sensitization of melanoma cells to TRAIL-induced apoptosis by 2-DG was studied by inhibition of the interaction between TRAIL and TRAIL-R2 using a TRAIL-R2/Fc chimeric protein. Figure 4A shows that the TRAIL-R2/Fc chimera significantly inhibited TRAIL-induced apoptosis in both Mel-RM and MM200 cells in the absence or presence of 2-DG (p < 0.05). Similarly, 2-DG-mediated sensitization of melanoma cells to TRAIL-induced apoptosis was blocked by either the general caspase inhibitor z-VAD-fmk, or the caspase-8 specific inhibitor z-IETD-fmk (p < 0.05) (Figure 4B & data not shown). In contrast, a TRAIL-R1/Fc chimeric protein displayed only minimal inhibitory effects on sensitization of Mel-RM and MM200 cells to TRAIL-induced apoptosis (Figure 4C).

To confirm the predominant role of up-regulation of TRAIL-R2 in sensitization of melanoma cells to TRAIL-induced apoptosis by 2-DG, we transfected a TRAIL-R2 specific siRNA pool into Mel-RM and MM200 cells. While TRAIL-R2 siRNA markedly inhibited TRAIL-R2 expression even in the presence of 2-DG, it inhibited TRAIL-induced apoptosis in the absence or presence of 2-DG (p < 0.05) (Figure 4D). Collectively, these results indicate that up-regulation of TRAIL-R2 on the cell surface is the main cause of sensitization of melanoma cells to TRAIL-induced apoptosis by 2-DG.

### 2-DG-mediated activation of TRAIL-R2 is independent of p53 and CHOP

**Figure 5:**
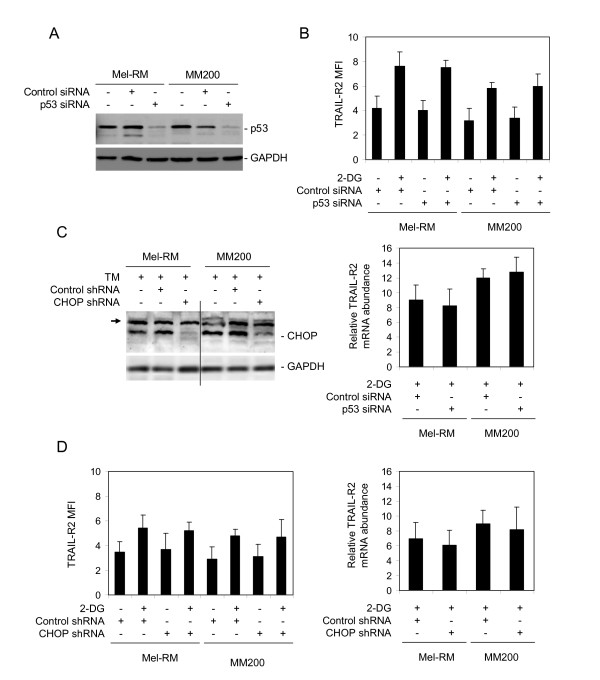
**2-DG-mediated up-regulation of TRAIL-R2 is independent of p53 and CHOP**. **A**, Mel-RM and MM200 cells were transfected with the control or p53 siRNA. Twenty-four hours later, whole cell lysates were subjected to Western blot analysis. **B**, Mel-RM and MM200 cells were transfected with the control or p53 siRNA. Twenty-four hours later, cells were treated with 2-DG (10 μM) for a further 16 hours. Upper panel: The cell surface expression of TRAIL-R2 was measured using flow cytometry. The data in y axes represent mean fluorescence intensity (MFI). Lower panel: Total RNA was isolated and subjected to Real-time PCR analysis for TRAIL-R2 mRNA expression. The relative abundance of mRNA expression before treatment was arbitrarily designated as 1. **C**, Whole cell lysates from Mel-RM and MM200 cells transduced with the control or CHOP shRNA were subjected to Western blot analysis. The arrow head points to non-specific bands generated by the antibody against CHOP. Note that the cells were treated with TM (3 μM) for 16 hours before harvest to better visualize CHOP. **D**, Mel-RM and MM200 cells transduced with the control or CHOP shRNA were treated with 2-DG (10 μM) for 16 hours. Left panel: The cell surface expression of TRAIL-R2 was measured using flow cytometry. The data in the y axes represent mean fluorescence intensity (MFI). Right panel: Total RNA was isolated and subjected to Real-time PCR analysis for TRAIL-R2 mRNA expression. The relative abundance of mRNA expression before treatment was arbitrarily designated as 1. The data shown are either the mean ± SE (B & D), or representative (A & C), of three individual experiments.

TRAIL-R2 is a transcriptional target of p53 [31]. However, up-regulation of TRAIL-R2 by 2-DG in the melanoma cell lines, ME4405 that lacks p53 expression [32] and Sk-Mel-28 that harbors mutated p53 [32], suggested that 2-DG-mediated up-regulation of TRAIL-R2 was independent of p53 (Figure 2B). To confirm this, we transfected a siRNA pool for p53 into Mel-RM and MM200 cells. As shown in Figure 5A, the cells transfected with the p53 siRNA, but not those with the control siRNA, displayed markedly lower levels of p53 expression. The reduced expression of p53 did not have any appreciable effect on 2-DG-mediated up-regulation of TRAIL-R2 on the cell surface and at the mRNA levels in both cell lines (Figure 5B).

Another transcription factor that is known to regulate TRAIL-R2 transcription in many cell types is CHOP [33,34]. We examined if CHOP contributes to 2-DG-mediated up-regulation of TRAIL-R2 in Mel-RM and MM200 cells with CHOP stably knocked down by lentiviral infections (Figure 5C). Deficiency in CHOP did not appear to significantly impact on the increase in TRAIL-R2 induced by 2-DG at both the protein and mRNA levels (Figure 5D). Together, these results indicate that neither p53 nor CHOP plays a role in 2-DG-mediated up-regulation of TRAIL-R2 in melanoma cells.

### 2-DG-mediated up-regulation of TRAIL-R2 is mediated by XBP-1

**Figure 6:**
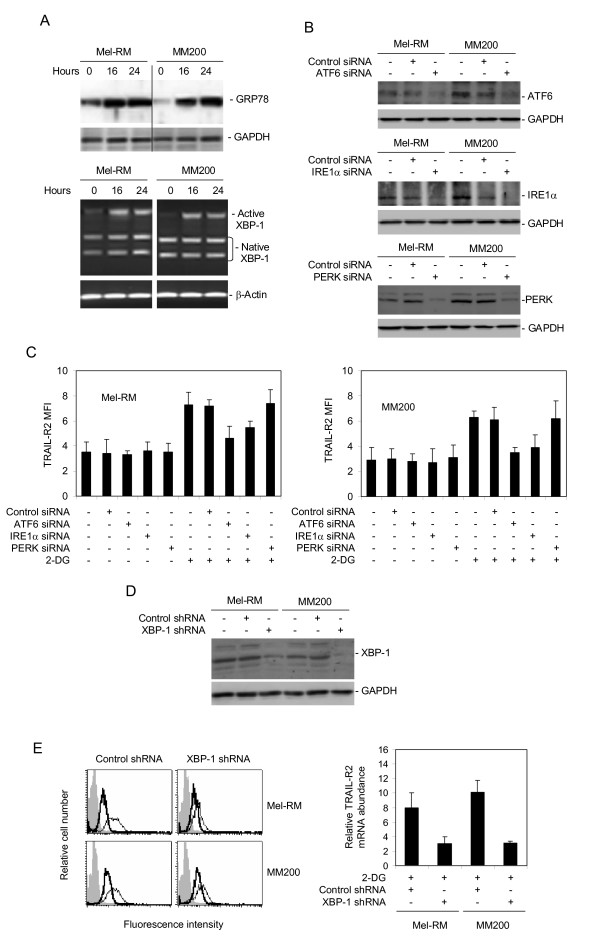
**XBP-1 plays an important role in up-regulation of TRAIL-R2 by 2-DG**. **A**, 2-DG activates the UPR in melanoma cells. Mel-RM and MM200 cells were treated with 2-DG (10 μM) for indicated periods. Upper panel: Whole cell lysates were subjected to Western blot analysis. Lower panel: RT-PCR products of XBP-1 mRNA from the cells were digested with Apa-LI for 90 minutes followed by electrophoresis. The longer fragment derived from the active form of XBP1 mRNA and two shorter bands derived from the inactive form are indicated. **B**, Mel-RM and MM200 cells were transfected with the control, IRE1α, ATF6, or PERK siRNA. Twenty-four hours later, whole cell lysates were subjected to Western blot analysis. **C**, Mel-RM (left panel) and MM200 (right panel) cells were transfected with the control, IRE1α, ATF6, or PERK siRNA. Twenty-four hours later, cells were treated with 2-DG (10 μM) for 24 hours. The cell surface expression of TRAIL-R2 was measured using flow cytometry. The data in the y axes represent mean fluorescence intensity (MFI). **D**, Whole cell lysates from Mel-RM and MM200 cells transduced with the control or XBP-1 shRNA were subjected to Western blot analysis. **E**, Left panel: Mel-RM and MM200 cells transduced with the control or XBP-1 shRNA were treated 2-DG (10 μM) for 24 hours. The cell surface expression of TRAIL-R2 was measured using flow cytometry. Filled histograms: isotype controls; Thick open histograms: TRAIL-R2 expression before treatment; Thin open histograms: TRAIL-R2 expression after treatment with 2-DG (10 μM) for 24 hours. Right panel: Total RNA from Mel-RM and MM200 cells transduced with the control or XBP-1 shRNA treated with 2-DG (10 μM) for 16 hours was isolated and subjected to Real-time PCR analysis for TRAIL-R2. The relative abundance of mRNA expression before treatment was arbitrarily designated as 1. Deficiency in XBP-1 significantly blocked up-regulation of TRAIL-R2 mRNA by 2-DG (p < 0.05). The data shown are either the mean ± SE (C & the right panel of E), or representative (A, B, D, & the left panel of E), of three individual experiments.

We have previously shown that the IRE1α and ATF6 pathways of the UPR are involved in transcriptional up-regulation of TRAIL-R2 by the classic ER stress inducers TM and TG [29,30]. We tested if 2-DG impinges on ER stress and activates the UPR in melanoma cells. As shown in Figure 6A, 2-DG up-regulated glucose-regulated protein 78 (GRP78) and the active form of x-box-binding protein-1 (XBP-1) mRNA, two commonly used markers of activation of the UPR [35,36].

To examine whether any of the UPR signaling pathways plays a role in up-regulation of TRAIL-R2 by 2-DG, we transfected siRNA pools for IRE1α, ATF6, and PERK into Mel-RM and MM200 cells, respectively (Figure 6B). As shown in Figure 6C, while the basal level of TRAIL-R2 expression was not impacted, up-regulation of TRAIL-R2 by 2-DG on the cell surface was partially inhibited in cells transfected with the siRNA for IRE1α and ATF6. In contrast, inhibition of PERK by siRNA did not alter the expression of TRAIL-R2 before and after treatment with 2-DG (Figure 6C).

The IRE1α and ATF6 signaling pathways of the UPR converge on the UPR effector XBP-1, as XBP-1 is transcriptionally regulated by ATF6, and its activation is mediated by IRE1α [25-27]. We therefore envisaged that XBP-1 plays a role in up-regulation of TRAIL-R2 by 2-DG in melanoma cells. To test this, we examined the effect of 2-DG on TRAIL-R2 expression in XBP-1-deficient melanoma cell lines established by stable knockdown with shRNA by lentiviral infections. Deficiency in XBP-1 inhibited 2-DG-induced up-regulation of TRAIL-R2 on the cell surface (Figures 6D &6E). Similarly, it blocked the increase in TRAIL-R2 transcription induced by 2-DG (Figures 6D &6E). Collectively, these results indicate that up-regulation of TRAIL-R2 by 2-DG is mediated by XBP-1 as a consequence of activation of the ATF6 and IRE1α pathways of the UPR.

### 2-DG up-regulates TRAIL-R2 and enhances TRAIL-induced apoptosis in fresh melanoma isolates

**Figure 7:**
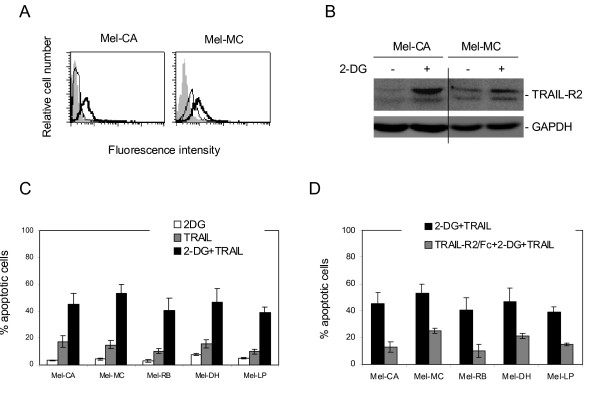
**2-DG up-regulates TRAIL-R2 and enhances TRAIL-induced apoptosis in fresh melanoma isolates**. **A**, Mel-CA and Mel-MC with (thick open histograms) or without (thin open histograms) treatment with 2-DG (10 μM) for 16 hours were subjected to measurement of the cell surface TRAIL-R2 expression in flow cytomety. The filled histograms are isotype controls. **B**, Whole cell lysates from Mel-CA and Mel-MC with or without treatment with 2-DG (10 μM) for 16 hours were subjected to Western blot analysis. **C**, Freshly isolated melanoma cells were treated with 2-DG (10 μM), TRAIL (200 ng/ml), or the combination of both for 24 hours were subjected to measurement of apoptosis by the propidium iodide method using flow cytometry. **D**, Freshly isolated melanoma cells were treated with a TRAIL-R2/Fc chimera (10 μg/ml) for 1 hour before the addition of 2-DG (10 μM) and TRAIL (200 ng/ml) for a further 24 hours. Apoptosis was measured by the propidium iodide method using flow cytometry. The data shown are either the mean ± SE (C & D), or representative (A & B), of three individual experiments.

Our previous studies have shown that fresh melanoma isolates, which may reflect more closely the in vivo situation, are relatively resistance to TRAIL-induced apoptosis due to low levels of expression of TRAIL death receptors [11]. We studied if 2-DG can also up-regulate TRAIL-R2 in fresh melanoma isolates. Freshly isolated melanoma cells, Mel-CA and Mel-MC were treated with 2-DG for 24 hours. As shown in Figures 7A and 7B, treatment with 2-DG increased the levels of TRAIL-R2 on the cell surface as measured in flow cytometry, and the TRAIL-R2 total protein levels as detected in Western blot analysis, in both Mel-CA and Mel-MC cells. Figure 7C shows that neither 2-DG nor TRAIL induced significant levels of apoptosis (< 20% apoptotic cells) in a panel of fresh melanoma isolates. However, co-treatment with 2-DG and TRAIL resulted in increases in the percentages of apoptotic cells (p < 0.05). Sensitization of fresh melanoma isolates to TRAIL-induced apoptosis by 2-DG was substantially inhibited by a recombinant TRAIL-R2/Fc chimera (p < 0.05) (Figure 7D), indicating that the effect of 2-DG on TRAIL-induced apoptosis in fresh melanoma isolates is largely accounted for by the increase in TRAIL-R2 expression on the cell surface.

## Discussion

The above results show that the combination of 2-DG and TRAIL, two promising anticancer agents, results in enhanced killing in cultured melanoma cell lines and fresh melanoma isolates. This is primarily due to up-regulation of TRAIL-R2 on the melanoma cell surface. Moreover, they demonstrate that 2-DG-mediated up-regulation of TRAIL-R2 is due to increased transcription, but this is not dependent on p53 and CHOP. Instead, the ATF6/IRE1α/XBP-1 axis of the UPR appears to play an important role in up-regulation of TRAIL-R2 induced by 2-DG in melanoma cells.

TRAIL is currently in clinical evaluation for the treatment of various cancers [8]. However, our past studies have shown that fresh isolates of melanoma and melanoma in tissue sections frequently had low TRAIL death receptor expression and therefore may be unresponsive to TRAIL [11,14]. Unlike studies in many other solid cancers, in which TRAIL-death receptors could be up-regulated by other clinically relevant therapeutic drugs [37-40], we have not found these to increase TRAIL death receptor expression in melanoma. Agents tested have included DNA-damaging agents, microtubulin-targeting agents, histone deacetylase inhibitors, and MEK inhibitors [[41], & data not shown]. Nevertheless, the classic ER stress inducers, the glycosylation inhibitor TM and the ER Ca^2+ ^ATPases inhibitor TG have been shown to enhance TRAIL-induced apoptosis in melanoma cells by up-regulation of TRAIL-R2 via activation of the UPR [29,30], but these compounds are not clinically applicable due to their toxicity towards normal tissues. The ability of 2-DG to up-regulate TRAIL death receptors in melanoma is therefore of particular interest, in that fluorodeoxyglucose is commonly used in clinical imaging, eg. positron emission tomography (PET) [42]. In addition, 2-DG alone or in combination with other therapeutics has been shown to inhibit tumor cell growth and has been in clinical trial for its potential as an anticancer agent [16,20-23].

Up-regulation of TRAIL death receptors by 2-DG was associated with enhanced apoptotic signaling induced by TRAIL. This was evidenced by increased activation of caspase-8, reduction in ΔΨm, mitochondrial release of cytochrome C, activation of caspase-3 and cleavage of its substrate PARP. Caspase-8 and -3 are the major initiator and effector caspase, respectively, in TRAIL-induced apoptosis of melanoma cells [2,3,10], whereas the mitochondrial apoptotic pathway is known to play an important role in TRAIL-induced apoptosis of melanoma [10,43]. In agreement with our previous finding that TRAIL-R2 is the dominant TRAIL death receptor in melanoma cells [9,10], inhibition of the interaction of TRAIL with TRAIL-R2, but not with TRAIL-R1, markedly blocked sensitization of melanoma cells to TRAIL-induced apoptosis by 2-DG, indicating that up-regulation of TRAIL-R2 was the main cause of sensitization of melanoma cells to TRAIL-induced apoptosis, even though both TRAIL-R1 and -R2 were increased by 2-DG. It is of note, however, the overall levels of TRAIL-R1 expression on the melanoma cell surface were lower than those of TRAIL-2 before and after treatment with 2-DG. Therefore, our results do not negate a potential role of TRAIL-R1 in mediating TRAIL-induced apoptosis in melanoma cells when it is expressed at relatively higher levels [44].

2-DG-mediated up-regulation of TRAIL-R2 on the melanoma cell surface was associated with elevated TRAIL-R2 total protein levels and increased TRAIL-R2 gene transcription. However, p53, which is known to mediate TRAIL-R2 transcription under many conditions [31,37], did not appear to play a part in up-regulation of TRAIL-R2 by 2-DG in melanoma cells. This was initially suggested by the finding that a p53-null melanoma cell line (ME4405), and a melanoma cell line carrying mutated p53 (Sk-Mel-28) displayed increased TRAIL-R2 in response to 2-DG. Further studies with siRNA knockdown of p53 in melanoma cell lines with wide-type p53 confirmed that inhibition of p53 did not impact on the up-regulation of TRAIL-R2 by 2-DG. These results, along with our previous observations that DNA-damaging agents such as cisplatin and adriamycin that increased the levels of p53 but did not up-regulate TRAL-R2 in melanoma cells [[32], & data not shown], suggest that p53 may not be functionally active in melanoma cells in regard to regulation of TRAIL-R2 expression. We have found that p53 in melanoma cells are frequently expressed as the smaller isoforms that aberrantly impact on the transcriptional activity of p53 [32].

We have previously shown that the ER stress inducers TM and TG could up-regulateTRAIL-R2 via the ATF6 and IRE1α pathways of the UPR independently of p53 [29,30]. In addition, the transcription factor CHOP that is an effector of the UPR also plays a part in up-regulation of TRAIL-R2 by TM and TG [29,30]. In this study, both the GRP78 protein and the active form of XBP-1 mRNA, two commonly used markers of activation of the UPR, were induced by 2-DG, indicating that, consistent with its inhibitory effect on glycolysis and glycosylation, 2-DG activated the UPR in melanoma cells. These results also suggest that the increase in TRAIL-R2 gene transcription might be the consequence of activation of UPR target genes. However, CHOP did not appear to contribute to increased TRAIL-R2 transcription, as deficiency in CHOP did not block up-regulation of TRAIL-R2 by 2-DG. It is unclear why CHOP played a role in up-regulation of TRAIL-R2 by TM and TG, but failed to do so in 2-DG-mediated up-regulation of TRAIL-R2, whereas all these compounds seemingly activated the UPR to comparable levels in melanoma cells [29,30]. A possible cause for this is that the cofactor(s) required by CHOP to trigger TRAIL-R2 transcription is not activated by 2-DG in melanoma cells. CHOP-mediated activation of Bim transcription is known to require the formation of CHOP-C/EBP heterodimers [45].

As with TM and TG, 2-DG-induced up-regulation of TRAIL-R2 in melanoma cells was partially inhibited by siRNA knockdown of IRE1α or ATF6, indicating that these pathways of the UPR are involved in up-regulation of TRAIL-R2 by 2-DG. Because XBP-1 is transcriptionally regulated by ATF6, and is activated by IRE1α [25-27], it seemed that XBP-1 may play a part in up-regulation of TRAIL-R2 mediated by these pathways of the UPR. In this study, deficiency in XBP-1 markedly blocked up-regulation of TRAIL-R2 in melanoma cells, verifying a role of XBP-1 in 2-DG-mediated up-regulation of TRAIL-R2. However, the UPR element (UPRE) or ER stress response element (ERSE) consensus sequence, which is characteristic of promoters of UPR target genes, could not be identified in the promoter region of the TRAIL-R2 gene (data not shown). This argues against a direct role of XBP-1 in activation of transcription of TRAIL-R2 in melanoma cells. It is conceivable that XBP-1 may activate TRAIL-R2 transcription indirectly via activation of an unknown transcription factor(s). Alternatively, XBP-1-mediated signaling may cause relief of transcriptional repression on the TRAIL-R2 promoter. In this regard, inactivation of the transcription repressor Yin Yang 1 (YY1) has been shown to lead to up-regulation of TRAIL-R2 in various types of cells [46]. Interestingly, YY1 is known to be regulated by O-Linked N-Acetylglucosaminylation (O-GlcNacylatiioioin), which was proposed to be connected with the pathway of glucose metabolism [47].

The finding that 2-DG could sensitize fresh melanoma isolates to TRAIL-induced apoptosis by up-regulation of TRAIL-R2 is of particular importance, for it is known that fresh melanoma isolates are relatively resistant to TRAIL-induced apoptosis due to low levels of TRAIL death receptor expression [11]. This may reflect more closely the in-vivo status of TRAIL death receptor expression in melanoma cells and their susceptibility to TRAIL-induced apoptosis. However, treatment with 2-DG also resulted in a small increase in TRAIL-R2 in normal cells such as melanocytes and fibroblasts, and caused increased toxicity towards the cells, suggesting that careful evaluation of low dose of 2-DG or its analogues in combination with low concentrations of TRAIL is required before investigations in patients are carried out.

## Conclusions

This study shows that 2-DG, a synthetic glucose analogue that inhibits glycolysis and glycosylation, up-regulates TRAIL death receptors and enhances TRAIL-induced apoptosis in cultured human melanoma cell lines and fresh melanoma isolates. Moreover, the study demonstrates that 2-DG-induced up-regulation of TRAIL-R2 is mediated by the ATF6/IRE1α/XBP-1 axis of the unfolded protein response independently of p53 and CHOP. Collectively, our data indicate that 2-DG is a promising agent to increase the therapeutic response of melanoma to TRAIL.

## Methods

### Cell Lines

Human melanoma cell lines Mel-RM, MM200, IgR3, Mel-CV, Mel-FH, Sk-Mel-28, Sk-Mel-110, and ME4405, have been described previously [9]. They were cultured in DMEM containing 5% FCS (Commonwealth Serum Laboratories, Melbourne, Australia). The cultured human melanocyte line HEMn-MP was purchased from Banksia Scientific (Bulimba, Qld, Australia) and the cells were cultured in medium supplied by Clonetics (Edward Kellar, Vic., Australia). Human embryonic fibroblasts (FLOW 2000) were cultured in DMEM containing 5% FCS as described previously [30].

### Fresh Melanoma Isolates

Isolation of melanoma cells from fresh surgical specimens was carried out as described previously [11].

### Antibodies, Recombinant Proteins, and Other Reagents

2-DG was purchased from Sigma Chemical Co. (Castle Hill, Australia). It was dissolved in DMSO to make up a stock solution of 1 mM. Recombinant human TRAIL and the TRAIL-R2/Fc chimera were supplied by Genentech Inc. (San Francisco, CA). The mouse MAbs against TRAIL-R1, -R2, -R3, and -R4 were also supplied by Genentech Inc. (San Francisco, CA). The cell-permeable general caspase inhibitor Z-Val-Ala-Asp(OMe)-CH2F (z-VAD-fmk) and the caspase-8 specific inhibitor Z-lle-Glu(Ome)-Thr-Asp(Ome)-CH_2_F (z-IETD-fmk) were purchased from Calbiochem (La Jolla, CA). The rabbit polyclonal Abs against caspase-3 and -8 were from Stressgen (Victoria, BC, Canada). The rabbit polyclonal Ab against against cleaved form of PARP was from Cell Signaling Technology (Beverly, MA). The rabbit mAbs against GRP78, XBP-1, IRE1α, ATF6, PERK, and CHOP, were purchased from Santa Cruz Biotechnology (Santa Cruz, CA). Isotype control Abs used were the ID4.5 (mouse IgG2α) mAb against *Salmonella typhi *supplied by Dr. L. Ashman (Institute for Medical and Veterinary Science, Adelaide, Australia), the 107.3 mouse IgG1 MAb purchased from PharMingen (San Diego, CA), and rabbit IgG from Sigma Chemical Co (Castle Hill, Australia).

### Flow Cytometry

Immunostaining on intact and permeabilized cells was carried out as described previously [9,11]. Analysis was carried out using a Becton Dickinson (Mountain View, CA) FACScan flow cytometer.

### Apoptosis

Quantitation of apoptotic cells by measurement of sub-G1 DNA content using the propidium iodide (PI) method or by Annexin-V staining was carried out as described elsewhere [9].

### Mitochondrial Membrane Potential (ΔΨm)

Melanoma cells were seeded at 1 × 10^5 ^cells/well in 24-well plates and allowed to reach exponential growth for 24 hours before treatment. Changes in ΔΨm were studied by staining the cells with the cationic dye, JC-1, according to the manufacture's instructions (Molecular Probes, Eugene, OR) as described previously [12].

### Western Blot Analysis

Western blot analysis was carried out as described previously [29,30]. Labeled bands were detected by Immun-Star™ HRP Chemiluminescent Kit, and images were captured and the intensity of the bands was quantitated with the Bio-Rad VersaDoc™ image system (Bio-Rad, Regents Park, NSW, Australia).

### Preparation of Mitochondrial and Cytosolic Fractions

Methods used for subcellular fraction were similar to the methods described previously [48].

### XBP-1 mRNA Splicin

The method used for detection of unspliced and spliced XBP-1 mRNAs was as described previously [28]. Briefly, RT-PCR products of XBP-1 mRNA were obtained from total RNA extracted using primers 5'-cggtgcgcggtgcgtagtctgga-3' (sense) and 5'-tgaggggctgagaggtgcttcct-3' (anti-sense). Because a 26 bp fragment containing an Apa-LI site is spliced upon activation of XBP-1 mRNA, the RT-PCR products were digested with Apa-LI to distinguish the active spliced form from the inactive unspliced form. Subsequent electrophoresis revealed the inactive form as two cleaved fragments and the active form as a non-cleaved fragment.

### Real-Time PCR

Quantitation of TRAIL-R2 mRNA expression using Real-Time PCR was performed as described previously. Briefly, total RNA was isolated, and reverse transcription PCR was carried out. The resulting cDNA products were used as templates for real-time PCR assays. Real-time PCR was performed using the ABI Prism 7700 sequence detection system (Applied Biosystems, Foster City, CA). For TRAIL-R2, Twenty-five μl mixtures were used for reaction, which contains 5 μl cDNA sample (0.5-1 μg/μl), 300 nM forward primers for TRAIL-R2 (CGCTGCACCAGGTGTGATT), 300 nM reverse primers for TRAIL-R2 (GTGCCGGCTTCGCACTGACA), 200 nM probes for TRAIL-R2 (6FAM-CCCTGCACCACGACCAGAAACACAG-TAMRA), and 9 mM MgCI_2_. Analysis of cDNA for β-actin was included as a control. The threshold cycle value (Ct) was normalized against β-actin cycle numbers. The relative abundance of mRNA expression of a control sample was arbitrarily designated as 1, and the values of the relative abundance of mRNA of other samples were calculated accordingly.

### Small RNA Interference (siRNA)

Melanoma cells were seeded at 3.5 × 10^4 ^cells/well in 24-well plates and allowed to reach approximately 50% confluence on the day of transfection. The siRNA constructs used were obtained as the siGENOME SMARTpool reagents (Dharmacon, Lafayette, CO). The siGENOME SMARTpool IRE1α (M-004951-01-0010), the siGENOME SMARTpool ATF6 (M-009917-01-0010), the siGENOME SMARTpool PERK (M-004883-01-0010), the siGENOME SMARTpool TRAIL-R2 (M-004819-01-0010), the siGENOME SMARTpool p53 (L-003329-00-0005), and the non-targeting siRNA control, SiConTRolNon-targeting SiRNA pool (D-001206-13-20) were obtained from Dharmacon. Cells were transfected with 50-100 nM siRNA in Opti-MEM medium (Invitrogen, Carlsbad, CA) with 5% fetal calf serum using Oligofectamine reagent (Invitrogen, Carlsbad, CA) according to the manufacturer's transfection protocol. Efficiency of siRNA was measured by Western blot analysis 24 hours after transfection.

### Short Hairpin RNA (shRNA) Knockdown

Melanoma cell lines were seeded at 1 × 10^4 ^per well in 96 well plates and left to attach overnight. Sigma MISSION^® ^Lentiviral Transduction Particles for shRNA-mediated knockdown of CHOP (SHVRS-NM-000100) and Sigma MISSION^® ^Lentiviral Transduction Particles for shRNA-mediated knockdown of XBP-1 (SHVRS-NM-000100)were applied to ~70% confluent cells in the presence of polybrene (4 or 8 μg/ml) at MOIs of 0.5, 1 or 5 in 100 μl DMEM. After 16 - 24 hours, the culture medium was replaced and cells were left another 24 hours. Cells were selected with 2 μg/ml puromycin for 3 days until mock-transduced controls (polybrene only) were completely dead. For each transduced melanoma cell line, up to 4 wells of cells per lentiviral clone were tested for knockdown via Western Analysis. Cells with lowest CHOP or XBP-1 levels were expanded for experimental use.

### Statistics

The significance of differences between experimental data was determined using the two-tailed student's t test for unpaired observations with Microsoft Excel 2000 software.

## Abbreviations

TRAIL: tumor necrosis factor-related apoptosis-inducing ligand; TRAIL-R: TRAIL receptor; 2-DG: 2-Deoxy-D-glucose; ER: endoplasmic reticulum; UPR: the unfolded protein response; IRE1α: inositol-requiring transmembrane kinase and endonuclease 1α; ATF6: activation of transcription factor 6; PERK: protein kinase-like ER kinase; XBP-1: x-box-binding protein-1. CHOP: CCAAT/enhancer-binding protein-homologous protein; GRP78: glucose-regulated protein 78; DMSO: Dimethyl Sulfoxide; MAb: monoclonal antibody; Ab: antibody; DISC: death-inducing signaling complex; FADD: Fas-associated death domain; TM: tunicamycin; TG: thapsigargin; ΔΨm: mitochondrial membrane potential; siRNA: small RNA interference; shRNA: Short hairpin RNA.

## Competing interests

The authors declare that they have no competing interests.

## Authors' contributions

HL, CCJ, CJL, AC, HYT, FY, and KHT performed the experiments, and analyzed the experiments results. PH and XDZ conceived the study, designed experiments, interpreted the findings and drafted the manuscript. All authors read and approved the final manuscript.

## References

[B1] Nagata S Apoptosis by death factor Cell 1997 88 355 365 10.1016/S0092-8674(00)81874-7 9039262

[B2] Ashkenazi A Dixit VM Death receptors: signaling and modulation Science 1998 281 1305 1308 10.1126/science.281.5381.1305 9721089

[B3] Walczak H Krammer PH The CD95 (APO-1/Fas) and the TRAIL (APO-2L) apoptosis systems Exp Cell Res 2000 256 58 66 10.1006/excr.2000.4840 10739652

[B4] Walczak H Miller RE Ariail K Gliniak B Griffith TS Kubin M Chin W Jones J Woodward A Le T Smith C Smolak P Goodwin RG Rauch CT Schuh JC Lynch DH Tumoricidal activity of tumor necrosis factor-related apoptosis-inducing ligand in vivo Nat Med 1999 5 157 63 10.1038/5517 9930862

[B5] Ashkenazi A Pai RC Fong S Leung S Lawrence DA Marsters SA Blackie C Chang L McMurtrey AE Hebert A DeForge L Koumenis IL Lewis D Harris L Bussiere J Koeppen H Shahrokh Z Schwall RH Safety and antitumor activity of recombinant soluble Apo2 ligand J Clin Invest 1999 104 155 62 10.1172/JCI6926 10411544 PMC408479

[B6] Hotte SJ Hirte HW Chen EX Siu LL Le LH Corey A Iacobucci A MacLean M Lo L Fox NL Oza AM A phase 1 study of mapatumumab (fully human monoclonal antibody to TRAIL-R1) in patients with advanced solid malignancies Clin Cancer Res 2008 14 3450 5 10.1158/1078-0432.CCR-07-1416 18519776

[B7] Plummer R Attard G Pacey S Li L Razak A Perrett R Barrett M Judson I Kaye S Fox NL Halpern W Corey A Calvert H de Bono J Phase 1 and pharmacokinetic study of lexatumumab in patients with advanced cancers Clin Cancer Res 2007 13 6187 94 10.1158/1078-0432.CCR-07-0950 17947486

[B8] Ashkenazi A Directing cancer cells to self-destruct with pro-apoptotic receptor agonists Nat Rev Drug Discov 2008 7 1001 12 10.1038/nrd2637 18989337

[B9] Zhang XD Franco A Myers K Gray C Nguyen T Hersey P Relation of TNF- related apoptosis-inducing ligand (TRAIL) receptor and FLICE inhibitory protein expression to TRAIL-induced apoptosis of melanoma Cancer Res 1999 59 2747 53 10364001

[B10] Hersey P Zhang XD How melanoma cells evade TRAIL-induced apoptosis Nat Rev Cancer 2001 1 142 50 10.1038/35101078 11905805

[B11] Nguyen DT Zhang XD Hersey P Relative resistance of fresh isolates of melanoma to tumor necrosis factor-related apoptosis-inducing ligand (TRAIL)-induced apoptosis Clin Cancer Res 2001 7 966s 73s 11300498

[B12] Zhang XD Wu JJ Gillespie SK Borrow JM Hersey P Human Melanoma Cells Selected for Resistance to Apoptosis by Prolonged Exposure to TRAIL are more Vulnerable to Non-Apoptotic Cell Death Induced by Cisplatin Clin Cancer Res 2006 12 1335 64 10.1158/1078-0432.CCR-05-2084 16489094

[B13] Wu JJ Zhang XD Gillespie S Hersey P Selection for TRAIL Resistance Results in Melanoma Cells with High Proliferative Potential FEBS Lett 2005 579 1940 4 10.1016/j.febslet.2005.02.041 15792800

[B14] Zhuang L Lee CS Scolyer RA McCarthy SW Zhang XD Thompson JF Screaton G Hersey P Progression in melanoma is associated with decreased expression of death receptors for tumor necrosis factor-related apoptosis-inducing ligand (TRAIL) Human Pathology 2006 37 1286 94 10.1016/j.humpath.2006.04.026 16949935

[B15] Warburg O On the origin of cancer cells Science 1956 123 309 14 10.1126/science.123.3191.309 13298683

[B16] Pelicano H Martin DS Xu RH Huang P Glycolysis inhibition for anticancer treatment Oncogene 2006 25 4633 46 10.1038/sj.onc.1209597 16892078

[B17] Hersey P Watts R Zhang XD Hackett J Metabolic Approaches to Treatment of Melanoma Clin Cancer Res 2009 15 6490 4 10.1158/1078-0432.CCR-09-0251 19861452

[B18] Weindruch R Keenan KP Carney JM Fernandes G Feuers RJ Floyd RA Halter JB Ramsey JJ Richardson A Roth GS Spindler SR Caloric restriction mimetics: metabolic interventions J Gerontol A Biol Sci Med Sci 2001 1 20 33 10.1093/gerona/56.suppl_1.20 12088209

[B19] Little E Ramakrishnan M Roy B Gazit G Lee AS The glucose-regulated proteins (GRP78 and GRP94): functions, gene regulation, and applications Crit Rev Eukaryot Gene Expr 1994 4 1 18 7987045 10.1615/critreveukargeneexpr.v4.i1.10

[B20] Kang HT Hwang ES 2-Deoxyglucose: an anticancer and antiviral therapeutic, but not any more a low glucose mimetic Life Sci 2000 78 1392 9 10.1016/j.lfs.2005.07.001 16111712

[B21] Liu H Hu YP Savaraj N Priebe W Lampidis TJ Hypersensitization of tumor cells to glycolytic inhibitors Biochemistry 2001 40 5542 7 10.1021/bi002426w 11331019

[B22] Lampidis TJ Kurtoglu M Maher JC Liu H Krishan A Sheft V Szymanski S Fokt I Rudnicki WR Ginalski K Lesyng B Priebe W Efficacy of 2-halogen substituted D-glucose analogs in blocking glycolysis and killing "hypoxic tumor cells" Cancer Chemother Pharmacol 2006 58 725 34 10.1007/s00280-006-0207-8 16555088

[B23] Maschek G Savaraj N Priebe W Braunschweiger P Hamilton K Tidmarsh GF De Young LR Lampidis TJ 2-deoxy-D-glucose increases the efficacy of adriamycin and paclitaxel in human osteosarcoma and non-small cell lung cancers in vivo Cancer Res 2004 64 31 4 10.1158/0008-5472.CAN-03-3294 14729604

[B24] Zhong D Xiong L Liu T Liu X Liu X Chen J Sun SY Khuri FR Zong Y Zhou Q Zhou W The Glycolytic Inhibitor 2-Deoxyglucose Activates Multiple Prosurvival Pathways through IGF1R J Biol Chem 2009 284 23225 33 10.1074/jbc.M109.005280 19574224 PMC2749096

[B25] Harding HP Calfon M Urano F Novoa I Ron D Transcriptional and translational control in the Mammalian unfolded protein response Annu Rev Cell Dev Biol 2002 18 575 99 10.1146/annurev.cellbio.18.011402.160624 12142265

[B26] Zhang K Kaufman RJ Signaling the unfolded protein response from the endoplasmic reticulum J Biol Chem 2004 18 279 25935 8 10.1074/jbc.R400008200 15070890

[B27] Schroder M Kaufman RJ The mammalian unfolded protein response Annu Rev Biochem 2005 74 739 89 10.1146/annurev.biochem.73.011303.074134 15952902

[B28] Jiang CC Chen LH Gillespie S Wang YF Kiejda KA Zhang XD Hersey P Inhibition of MEK sensitizes human melanoma cells to endoplasmic reticulum stress-induced apoptosis Cancer Res 2007 67 9750 61 10.1158/0008-5472.CAN-07-2047 17942905

[B29] Chen LH Jiang CC Kiejda KA Wang YF Thorne RF Zhang XD Hersey P Thapsigargin sensitizes human melanoma cells to TRAIL-induced apoptosis by up-regulation of TRAIL-R2 through the unfolded protein response Carcinogenesis 2007 28 2328 36 10.1093/carcin/bgm173 17652336

[B30] Jiang CC Chen LH Gillespie S Kiejda KA Mhaidat N Wang YF Thorne R Zhang XD Hersey P Tunicamycin sensitizes human melanoma cells to tumor necrosis factor-related apoptosis-inducing ligand-induced apoptosis by up-regulation of TRAIL-R2 via the unfolded protein response Cancer Res 2007 67 5880 8 10.1158/0008-5472.CAN-07-0213 17575157

[B31] Wu GS Burns TF McDonald ER Jiang W Meng R Krantz ID Kao G Gan DD Zhou JY Muschel R Hamilton SR Spinner NB Markowitz S Wu G el-Deiry WS KILLER/DR5 is a DNA damage-inducible p53-regulated death receptor gene Nat Genet 1997 17 141 3 10.1038/ng1097-141 9326928

[B32] Avery-Kiejda KA Zhang XD Adams LJ Scott RJ Vojtesek B Lane DP Hersey P Small molecular weight variants of p53 are expressed in human melanoma cells and are induced by the DNA-damaging agent cisplatin Clin Cancer Res 2008 14 1659 68 10.1158/1078-0432.CCR-07-1422 18310316

[B33] Yamaguchi H Wang HG CHOP is involved in endoplasmic reticulum stress-induced apoptosis by enhancing DR5 expression in human carcinoma cells J Biol Chem 2004 279 45495 502 10.1074/jbc.M406933200 15322075

[B34] Yoshida T Shiraishi T Nakata S Horinaka M Wakada M Mizutani Y Miki T Sakai T Proteasome inhibitor MG132 induces death receptor 5 through CCAAT/enhancer-binding protein homologous protein Cancer Res 2005 65 5662 7 10.1158/0008-5472.CAN-05-0693 15994939

[B35] Hirota M Kitagaki M Itagaki H Aiba S Quantitative measurement of spliced XBP1 mRNA as an indicator of endoplasmic reticulum stress J Toxicol Sci 2006 31 149 156 10.2131/jts.31.149 16772704

[B36] Lee AS The ER chaperone and signaling regulator GRP78/BiP as a monitor of endoplasmic reticulum stress Methods 2005 35 373 381 10.1016/j.ymeth.2004.10.010 15804610

[B37] Sheikh MS Burns TF Huang Y Wu GS Amundson S Brooks KS Fornace AJ Jr el-Deiry WS p53-dependent and -independent regulation of the death receptor KILLER/DR5 gene expression in response to genotoxic stress and tumor necrosis factor alpha Cancer Res 1998 58 1593 8 9563466

[B38] Singh TR Shankar S Chen X Asim M Srivastava RK Synergistic interactions of chemotherapeutic drugs and tumor necrosis factor-related apoptosis-inducing ligand/Apo-2 ligand on apoptosis and on regression of breast carcinoma in vivo Cancer Res 2003 63 5390 400 14500373

[B39] Nakata S Yoshida T Horinaka M Shiraishi T Wakada M Sakai T Histone deacetylase inhibitors upregulate death receptor 5/TRAIL-R2 and sensitize apoptosis induced by TRAIL/APO2-L in human malignant tumor cells Oncogene 2004 19 6261 71 10.1038/sj.onc.1207830 15208660

[B40] Chinnaiyan AM Prasad U Shankar S Hamstra DA Shanaiah M Chenevert TL Ross BD Rehemtulla A Combined effect of tumor necrosis factor-related apoptosis-inducing ligand and ionizing radiation in breast cancer therapy Proc Natl Acad Sci USA 2000 97 1754 9 10.1073/pnas.030545097 10677530 PMC26508

[B41] Gillespie S Borrow J Zhang XD Hersey P Bim plays a crucial role in synergistic induction of apoptosis by the histone deacetylase inhibitor SBHA and TRAIL in melanoma cells Apoptosis 2006 11 2251 65 10.1007/s10495-006-0283-6 17051334

[B42] Pauleit D Stoffels G Bachofner A Floeth FW Sabel M Herzog H Tellmann L Jansen P Reifenberger G Hamacher K Coenen HH Langen KJ Comparison of (18)F-FET and (18)F-FDG PET in brain tumors Nucl Med Biol 2009 36 779 87 10.1016/j.nucmedbio.2009.05.005 19720290

[B43] Zhang XD Zhang XY Gray CP Nguyen T Hersey P Tumor necrosis factor-related apoptosis-inducing ligand-induced apoptosis of human melanoma is regulated by smac/DIABLO release from mitochondria Cancer Res 2001 61 7339 48 11585775

[B44] Bae SI Cheriyath V Jacobs BS Reu FJ Borden EC Reversal of methylation silencing of Apo2L/TRAIL receptor 1 (DR4) expression overcomes resistance of SK-MEL-3 and SK-MEL-28 melanoma cells to interferons (IFNs) or Apo2L/TRAIL Oncogene 2008 27 490 8 10.1038/sj.onc.1210655 17653094

[B45] Puthalakath H O'Reilly LA Gunn P Lee L Kelly PN Huntington ND Hughes PD Michalak EM McKimm-Breschkin J Motoyama N Gotoh T Akira S Bouillet P Strasser A ER stress triggers apoptosis by activating BH3-only protein Bim Cell 2007 129 1337 49 10.1016/j.cell.2007.04.027 17604722

[B46] Huerta-Yepez S Vega M Escoto-Chavez SE Murdock B Sakai T Baritaki S Bonavida B Nitric oxide sensitizes tumor cells to TRAIL-induced apoptosis via inhibition of the DR5 transcription repressor Yin Yang 1 Nitric Oxide 2009 20 39 52 10.1016/j.niox.2008.08.001 18778787

[B47] Hiromura M Choi CH Sabourin NA Jones H Bachvarov D Usheva A YY1 is regulated by O-linked N-acetylglucosaminylation (O-glcNAcylation) J Biol Chem 2003 278 14046 52 10.1074/jbc.M300789200 12588874

[B48] Wang YF Jiang CC Kiejda KA Gillespie S Zhang XD Hersey P Apoptosis induction in human melanoma cells by inhibition of MEK is caspase-independent and mediated by the Bcl-2 family members PUMA, Bim, and Mcl-1 Clin Cancer Res 2007 13 4934 42 10.1158/1078-0432.CCR-07-0665 17652623

